# Leukocyte infiltration in experimental stroke

**DOI:** 10.1186/1742-2094-10-115

**Published:** 2013-09-18

**Authors:** Nina Vindegaard Grønberg, Flemming Fryd Johansen, Uffe Kristiansen, Henrik Hasseldam

**Affiliations:** 1 Department of Biomedical Sciences, University of Copenhagen, Ole Maaloesvej 5, Copenhagen 2200, Denmark; 2 Department of Drug Design and Pharmacology, University of Copenhagen, Universitetsparken 2, Copenhagen 2100, Denmark

**Keywords:** Leukocytes, Macrophages, Middle cerebral artery occlusion (MCAO), Neutrophil granulocytes, Stroke, T-cells

## Abstract

Stroke is one of the leading causes of death worldwide. At present, the only available treatment is thrombolysis, which should be initiated no later than 4.5 hours after onset of symptoms. Several studies have shown that an attenuation of the inflammatory response in relation to stroke could widen the therapeutic window. However, the immune system has important functions following infarction, such as removal of dead cells and the subsequent astrocytosis as well as prevention of post-ischemic infection. Hence, detailed knowledge concerning the temporal profile of leukocyte infiltration is necessary in order to develop new and effective treatments.

The purpose of this review is to determine the temporal profile of leukocyte (neutrophil granulocytes, macrophages and T-cells) infiltration following experimental stroke. We found that the number of neutrophil granulocytes peaks between day 1 and 3 after experimental stroke, with short occlusion times (30 and 60 minutes of middle cerebral artery occlusion (MCAO)) leading to a later peak in response (*P* <0.001). Macrophages/microglia were found to peak later than day 3 and stay in the infarcted area for longer time periods, whereas duration of occlusion had no influence on the temporal infiltration (*P* = 0.475). Studies on T-cell infiltration are few; however, a tendency towards infiltration peak at later time points (from day 4 onwards) was seen.

This review provides a framework for the instigation of post-stroke anti-inflammatory treatment, which could prove beneficial and widen the therapeutic window compared to current treatment options.

## Introduction

According to the World Health Organization (WHO), stroke and other cerebrovascular disease is the second leading cause of death worldwide [1]. Stroke is most often caused by vessel occlusion (85% of cases), where an infarct develops after a few minutes of ischemia [2]. At present, the only available treatment is the clot-busting drug tissue plasminogen activator (tPA), which must be administered no later than 4.5 hours after onset of symptoms [3] - a very narrow therapeutic window.

The area of infarction contains a core of dead neurons, which cannot be rescued, and the surrounding penumbra, containing degenerated but not yet dead neurons [4]. Within the core the cells undergo anoxic depolarization without subsequent repolarization, while in the penumbra repeated de- and repolarizations lead to elevated levels of extracellular glutamate. This elevation leads to increases in intracellular Na^+^ and Ca^2+^[5]. The elevated level of intracellular Ca^2+^ leads to an increased production of free radicals [2], furthermore leading to neuronal death, which then activates resident cells, such as microglia and astrocytes, whose inflammatory mediators further activate and attract leukocytes [6].

It is known that the inflammatory process has an impact on post-ischemic tissue damage in the brain [2,7-10]. In particular, neutrophil granulocytes (neutrophils) [11-13] and macrophages/microglia [7,14,15] have been regarded as the main players in post-ischemic infarction. However, recent studies have shown that T-cells also have an impact on tissue damage [4,16-20]. Several studies have shown that an attenuation of the inflammatory process could be a possible way to widen the therapeutic window [8,20,21]. However, other studies have pointed out that the inflammatory response is necessary in order, for example, to remove dead neurons and other brain cells in the ischaemic core, and promote astrocytosis [16,22].

Consequently, it is not possible to categorize the inflammatory process as being either beneficial or deleterious in relation to stroke. Given that the immune system consists of a variety of different cell populations with a range of different functions at different time points following tissue damage, detailed analyses of specific immune cell infiltration is certainly needed.

Therefore, the purpose of this review is to determine the temporal infiltration profile of neutrophil granulocytes, macrophages and T-cells as a consequence of experimental stroke. This could potentially enhance the development and implementation of drug candidates targeted against one or several immune cell population(s) at certain time points post-stroke, and thereby widen the therapeutic window.

## Materials and methods

A total of 19 studies were included in this review based on the following inclusion criteria: 1) the study had a clear presentation of data (time and amount of cells per given unit); 2) quantification was performed at a minimum of two different time points; and 3) permanent or transient middle cerebral artery occlusion (pMCAO or tMCAO) were performed on rats or mice.

The exclusion criteria were: 1) the study was based on experiments with rats or mice suffering from another disease, since some diseases seem to have an influence on the infarct size [23]; 2) the study was based on experiments with neonatal rats or mice; and 3) the study was based on experiments with female rats or mice, or the gender of the animals was not specified, since it has been shown that gender has an influence on the infarct size post-stroke in rats [24]. 4) The experimental research of the study should be carried out in order to internationally recognized guidelines.

The included studies used different ways to quantify the amount of leukocytes after MCAO (for example, number of cells per hemisphere, 40 × field, and so on). To be able to compare data we normalized the amount of cells in a given study to the highest amount of cells found in that particular study. Thus, all data are presented as the percentage of the highest amount of cells within the specific experiment. It should be noted that the highest amount of cells in a given study does not necessarily reflect the true peak of cell infiltration and could lead to ambiguous conclusions, since the included studies did not investigate the amount of cells at all time points.

Some papers [25-28] quantified cells in different areas of the brain (cortex, striatum and preoptic area). Thus, we estimated the volume of these three areas by using stereotaxic atlases [29,30] and weighted the data accordingly (Table 1). The studies in which weighted averages were calculated are marked (^Δ^) in Tables 2 and 3.

**Table 1:** Volume of cortex, striatum and preoptic area in rat and mouse brain

**Animal**	**Cortex (mm**^**3**^**)**	**Striatum (mm**^**3**^**)**	**Preoptic area (mm**^**3**^**)**
Rat	610	98	2.4
Mouse	35	10	0.3

**Table 2:** Amount of neutrophils (normalized) at different time points post-ischemia listed according to duration of occlusion (tMCAO and pMCAO)

**Occlusion time**	**Amount of neutrophils (survival time)**										**Strain**	**n**	**Marker**	**Reference**
	**4 hours**	**6 hours**	**12 hours**	**18 hours**	**1 day**	**2 days**	**3 days**	**4 days**	**5 days**	**7 days**				
30 minutes					-	++		**+++**		+++	C57Bl/6	4	Anti-neutro	[31]
30 minutes					-	+		**+++**		+	C57Bl/6	4	Anti-neutro	[32]
60 minutes	+				+	++	+++				C57Bl/6	6	NIMP-R14	[25]
60 minutes	-				++		+++				C57Bl/6	5	PMN	[33]
60 minutes				-	+	++	+++	++			C57Bl/6 J	7	Lγ-6G	[34]
60 minutes						+++	++		+	-	Wistar	4	MPO	[35]
60 minutes		-	++		++		+++			-	Wistar	6	MPO	[36]
120 minutes			++		+++		+				Wistar	8	MPO	[37]
120 minutes					+++			-		-	Sprague–Dawley	5	H&E	[38]
120 minutes^Δ^		+	++		+++	+++	+	-		-	Wistar	4	H&E	[28]
pMCAO^Δ^			-		+	+	+++	+		-	Wistar	6	H&E	[26]
pMCAO^Δ^		-	-		+	+++	++	-		-	Wistar	5	H&E	[28]

Crosses represent the following amount of cells presented as percentage of the highest cell number: +, 25 to 49%; ++, 50 to 94%; +++, 95 to 100%; -, <25%. ^Δ^Weighted average. H&E, hematoxylin and eosin; MPO, myeloperoxidase; n, number of animals included; neutro, neutrophil granulocyte; pMCAO, permanent middle cerebral artery occlusion; PMN, polymorphonuclear; tMCAO, transient middle cerebral artery occlusion.

**Table 3:** Amount of macrophages/microglia (normalized) at different time points post- ischemia listed according to duration of occlusion (tMCAO and pMCAO)

**Occlusion time**	**Amount of macrophages/microglia (days of survival)**										**n**	**Strain**	**Marker**	**Reference**
	**<1**	**1**	**2**	**3**	**4**	**5**	**7**	**14**	**21**	**≥30**				
30 minutes^Δ^	++	++	++	+++							5	C57Bl/6	Iba-1	[25]
30 minutes		+	++		+++		++				4	C57Bl/6	F4/80	[31]
30 minutes		+	++		+++		++				4	C57Bl/6	F4/80	[32]
60 minutes^Δ^	++	++	++	+++							6	C57Bl/6	Iba-1	[25]
60 minutes^Δ^		+		+++			++				5	C57Bl/6	Iba-1	[27]
120 minutes	+	+		+++							8	Wistar	CD68	[37]
120 minutes		-		++			+++				7	Sprague–Dawley	CD68	[39]
pMCAO^Δ^	-	-	-	++	+++	++					6	Wistar	H&E	[26]
pMCAO				+			++	+++	++	++	6	Sprague–Dawley	CD68	[40]
pMCAO		+		+++			+++	+++		++	5	Wistar and Lewis	CD68	[41]

Crosses represent the following amount of cells presented as percentage of the highest cell number: +, 25 to 49%; ++, 50 to 94%; +++, 95 to 100%; -, <25%. ^Δ^Weighted average. H&E, hematoxylin and eosin; n, number of animals included; pMCAO, permanent middle cerebral artery occlusion; tMCAO, transient middle cerebral artery occlusion.

In all the included studies, ischemia was induced by inserting a filament in the common carotid artery, except Mu *et al*. [40] and Schroeter *et al*. [41] (electrocoagulation and forceps, respectively).

## Results

### Temporal infiltration of neutrophils

**Figure 1:**
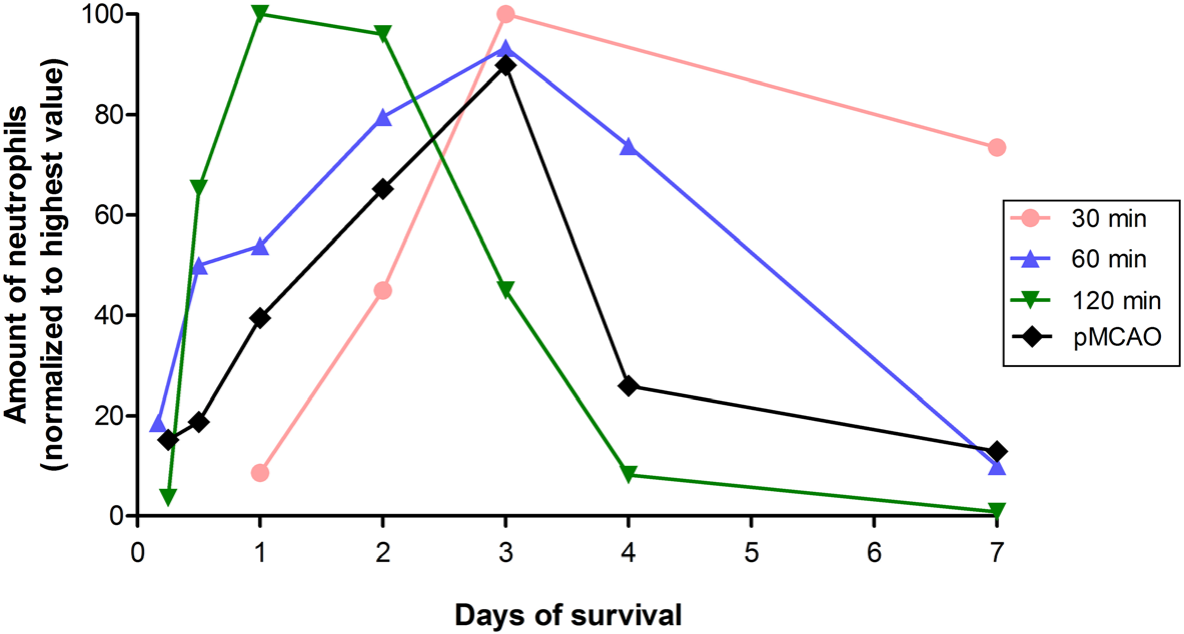
**Mean of normalized amount of infiltrating neutrophils according to survival time.** Duration of occlusion has an influence on the temporal profile of neutrophilic infiltration, suggesting that 120 minutes of MCAO results in an early massive influx of neutrophil granulocytes compared to the shorter occlusion times. Data are also presented in Table 2. MCAO, middle cerebral artery occlusion; pMCAO, permanent middle cerebral artery occlusion.

The infiltration of neutrophils follows a relatively narrow temporal profile, both after tMCAO including reperfusion and pMCAO, with a maximum between day 1 and 3 (Figure 1 and Table 2).

Data indicated that the occlusion time has an influence on the subsequent temporal profile of the infiltration of neutrophils. Thus, a two-way analysis of variance (ANOVA) was performed to test if there was an interaction between duration of occlusion and the relative number of cells at different survival times (with pMCAO considered as infinite time of occlusion). This showed that the temporal profile of neutrophil infiltration depends on the duration of occlusion (*P* <0.001). Consequently, multiple pairwise comparisons were performed (Holm-Sidak method, Table 4).

**Table 4:** Pairwise multiple comparisons (Holm-Sidak method) of amount of neutrophils at different time points of survival and occlusion

**Comparison for factor**	**Comparison**	**Unadjusted *P***	**Level of significance**
Survival time (30 minutes of occlusion)	3 days versus 1 day	<0.001	**
	7 days versus 1 day	0.004	*
	3 days versus 2 days	<0.012	*
Survival time (60 minutes of occlusion)	3 days versus 7 days	<0.001	**
	2 days versus 7 days	<0.001	**
	3 days versus 1 day	0.008	*
Survival time (120 minutes of occlusion)	1 day versus 7 days	<0.001	**
	2 days versus 7 days	<0.001	**
	1 day versus 3 days	0.007	*
Survival time (pMCAO)	3 days versus 7 days	<0.001	**
Occlusion time (1 day of survival)	120 minutes versus 30 minutes	<0.001	**
	120 minutes versus pMCAO	0.003	*
	120 minutes versus 60 minutes	0.006	*
	60 minutes versus 30 minutes	0.016	*
Occlusion time (7 days of survival)	30 minutes versus 120 minutes	0.001	**
	30 minutes versus 60 minutes	0.004	*
	30 minutes versus pMCAO	0.006	*

*P <0.05; **P <0.01. pMCAO, permanent middle cerebral artery occlusion.

A survival time of 30 and 60 minutes of occlusion resulted in a steady increase in the quantity of neutrophils between day 1 and 3 of survival (Figure 1 and Table 2). This is in contrast to 120 minutes of occlusion, which resulted in a more acute infiltration, peaking after 24 hours, followed by a decline. This is most likely due to massive vascular destruction as well as more pronounced tissue damage after 120 minutes, resulting in high amounts of cellular debris and thus chemotaxis.

Regarding pMCAO, the neutrophilic infiltration was more or less similar to 30 and 60 minutes of occlusion, most likely signifying a more massive tissue damage with a restricted cellular influx (Figure 1).

### Temporal infiltration of macrophages and microglia

**Figure 2:**
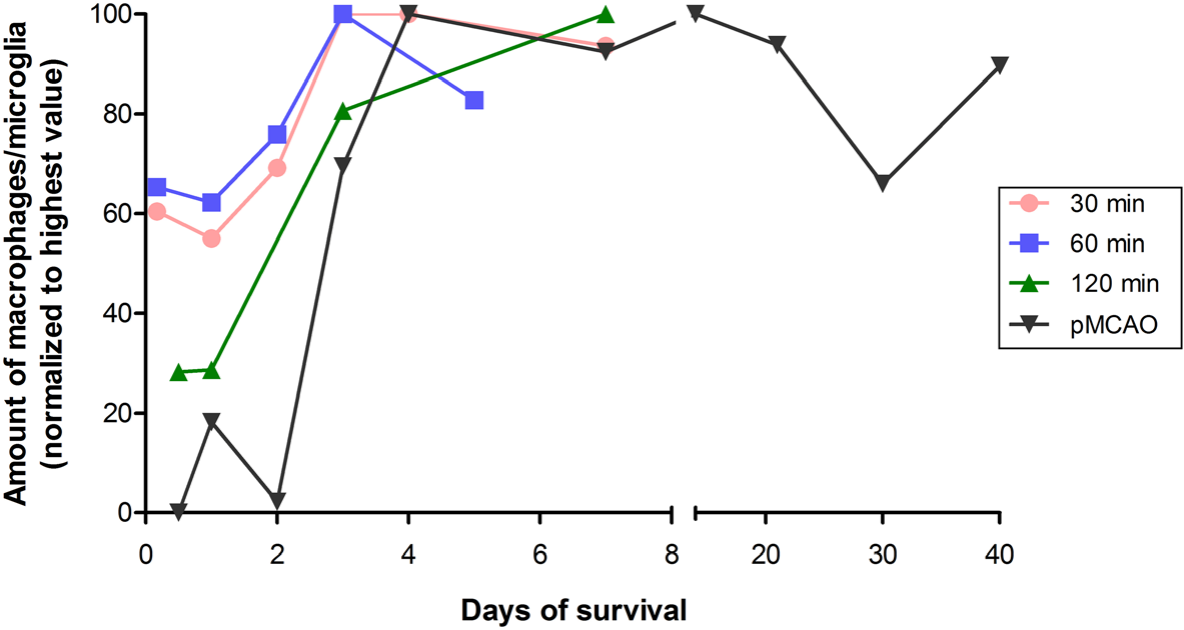
**Mean of normalized amount of infiltrating macrophages/microglia according to survival time.** The amount of macrophages/microglia in the infarcted area increases until day 4 to 7 after experimental stroke, whereafter a plateau is reached. Data are also presented in Table 3. pMCAO, permanent middle cerebral artery occlusion.

The presence of macrophages/microglia in the post-ischemic brain exhibits a different pattern as compared to neutrophils. Whereas the amount of neutrophils declined after day 3, the macrophages/microglia resided in the tissue for substantially longer time periods. The infiltration seemed to peak between day 3 and 7; however, many of the included studies did not investigate cellular influx beyond day 7 (Figure 2 and Table 3). Nevertheless, a significant increase in cellular infiltration from day 1 to days 4 and 7 was seen (ANOVA, *P* <0.001) after allowing for differences in occlusion time. No interaction between occlusion time and temporal infiltration was seen (two-way ANOVA, *P* = 0.475), suggesting that the amount of macrophages/microglia post-stroke is not influenced by the duration of the vascular occlusion. Yet, the sparse amount of data resulted in a very weak power (occlusion time: 1-β = 0.249; occlusion time x survival time: 1-β = 0.05 with α = 0.05).

### Temporal infiltration of T-cells

**Figure 3:**
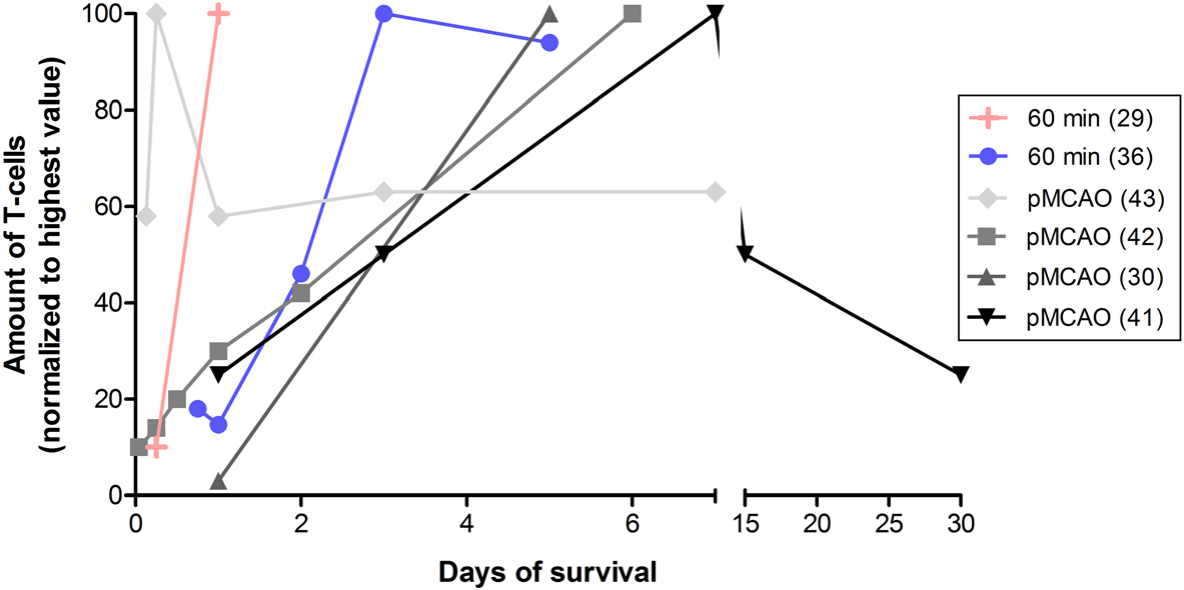
**Mean of normalized amount of infiltrating T-cells according to survival time.** Infiltration peaks around day 5, followed by a decline up until day 30 (according to Schroeter *et al*. [41]). Data are also presented in Table 5.

Only five studies were found that quantified total T-cell influx following stroke by using the pan T-cell marker CD3 (Table 5). One study used CD5 as a marker for T-cells; however, this molecule is expressed by subsets of B-cells as well. Nevertheless, T-cell infiltration after 60 minutes of MCAO peaks around day 3, whereas infiltration peaks at around day 5 to 7 following pMCAO (Figure 3).

**Table 5:** Amount of T-cells (CD3 and CD5), T-helper cells (CD4), cytotoxic T-cells (CD8) and B-cells (B220) (normalized) at different time points post-ischemia

**Occlusion time**	**Amount of lymphocytes (days of survival)**										**Marker**	**n**	**Strain**	**Reference**
	**<1**	**1**	**2**	**3**	**4**	**5**	**6**	**7**	**14**	**≥30**				
60 minutes	-	+++									CD3	6	C57Bl/6	[42]
60 minutes	-	-	+	+++	++						CD3	6	C57Bl/6	[34]
pMCAO		-				+++					CD3	8	C57Bl/6	[43]
pMCAO	+++	++		++				++			CD3	ns	CB17	[44]
pMCAO	-	+	+				+++				CD3	6	Sprague–Dawley	[45]
pMCAO		+		++				+++	++	+	CD5	5	Wistar	[41]
120 minutes	+++	++		+							CD4	8	Wistar	[37]
pMCAO	++	++		++					++		CD4	ns	CB17	[44]
120 minutes	-	-		+++							CD8	8	Wistar	[37]
pMCAO			+		++			+++	++	-	CD8	5	Wistar	[41]
60 minutes		-	-	-	+	+++					B220	6	C57Bl/6	[34]
pMCAO			-			+++					B220	8	C57Bl/6	[43]

Crosses represent the following amount of cells presented as the percentage of the highest cell number: +, 25 to 49%; ++, 50 to 94%; +++, 95 to 100%; -,<25%. n, number of animals included; ns, not shown; pMCAO, permanent middle cerebral artery occlusion.

Data are, however, scarce and inconsistent. Takata *et al*. [44] found that the amount of T-cells peaked 6 hours after pMCAO, while the other pMCAO studies observed T-cells peaking at later time points [34,41,45]. A couple of studies investigated the influx of T-helper (Th) and cytotoxic T-cell subsets following stroke (Table 5). Here, a tendency towards an early peak (day 1) in CD4^+^ T-cell infiltration and a later peak (day 3 to 7) in CD8^+^ T-cell infiltration was seen. Two studies quantified the amount of B-cells (B220^+^) post-stroke and found that infiltration peaks at day 5 or later (Table 5).

## Discussion

### Significance of neutrophilic influx

Matsuo *et al*. [12] and Satoh *et al*. [13] have shown that neutrophils are the main source of free oxygen radicals post-stroke, which directly destroy the neurons. Barone *et al*. [11] have shown that neutrophils are harmful due to their endothelial damaging properties, while Rosell *et al*. [46] found an association between the destruction of a structural component of the blood–brain barrier and the infiltration of matrix metalloproteinase (MMP)-9-positive neutrophils in humans. Based on this, it is likely that treatment targeted against neutrophils will reduce the post-ischemic brain damage, as Iadecola *et al*. discuss in their review [8]. Furthermore, McCarter *et al*. [47] have shown that FK-506 reduces the amount of tissue damage following experimental stroke due to inhibition of neutrophilic infiltration. In contrast, Harris *et al*. [48] conclude that neutrophils have no influence on infarct size during the acute phase post-stroke and Fassbender *et al*. [49] have shown that an influx of neutrophils six times the normal amount had no influence on infarct size. In addition to this, various treatment strategies (anti-intercellular adhesion molecule 1 (ICAM-1) antibody, anti-CD18 antibody, neutrophil inhibitory factor and IL-1 receptor antagonist) aimed at reducing the neutrophilic infiltration have shown no effect on outcome in clinical trials involving stroke patients [50]. These conflicting results could be explained by the temporal differences in neutrophilic influx, as presented in this review (Figure 1 and Tables 2 and 4). Future trials should take these differences into account, and design the therapy according to time frame and stroke severity.

### Significance of macrophage/microglial infiltration

Regarding macrophage/microglial infiltration into the affected hemisphere, we did not find an interaction between occlusion time and survival time. However, the amount of cells significantly increased over time, until a plateau was reached around day 4, independent of occlusion time (Figure 2).

The fact that 30 and 60 minutes of occlusion only resulted in less than a doubling of the amount of macrophages/microglia (from approximately 60 to 100%) could reflect the less severe damage following the short occlusion times; less tissue damage results in a lower cell density. The huge increase in cell infiltration following 120 minutes of occlusion and pMCAO could also, at least partially, be due to a more massive microglial death initially, followed by a massive influx of macrophages.

It is known that macrophages/microglia excrete reactive oxygen species (ROS) and NO, which is toxic to the brain tissue following an ischemic insult [6]. However, microglia also has neurotrophic properties, such as production of neurotrophins and brain-derived neurotrophic factor (BDNF) [6]. Clark *et al*. [51] found that even though doxycycline (a drug that inhibits leukocyte adhesion) reduces the infarct size, it does not reduce the amount of macrophages/microglia. This could indicate that the main function of these cells is to phagocytose and remove the damaged brain tissue instead of destroying viable cells. However, it should be kept in mind that macrophages and microglia, like neutrophils, have the ability to recruit and activate other parts of the immune system, such as lymphocytes.

It should be noted that Matsumoto *et al*. [52] found that CD68 is expressed on neutrophils as well. However, even if this is the case, the purpose of this review is to provide an overview of published studies that investigate immune cell influx at various time points following MCAO. In all the studies included, CD68 is used as a marker for macrophages and microglia.

### Temporal infiltration of T-cells and their subtypes

Only a few studies examined T-cells and their various subtypes in relation to post-ischemic infiltration (Table 5). As shown in Figure 3, there was a tendency towards a T-cell peak no earlier than day 5. The study by Takata *et al*. [44] found that T-cells peak 6 hours after pMCAO is initiated, but this is not in line with the other studies. Schroeter *et al*. [41] stands for the most solid investigation of the temporal profile of T-cell infiltration and found a peak at day 7, followed by a decline up until day 30 (latest time point investigated). However, these data need to be interpreted with caution given that they stained for CD5, which in addition to T-cells is present on a subset of non-adaptive B-cells, called B-1 cells.

Several authors suggested that T-cells are harmful post-stroke [16,17], whereas B-cells have been shown to play a minor role [18,20]. Based on several studies of mice lacking T-cells, Brait *et al*. [4] concluded that the lack of T-cells reduces infarct size. In line with this, various T-cell-specific therapies, such as anti-α4 integrin antibodies and vascular cell adhesion molecule 1 (VCAM-1) siRNA, which inhibit T-cell infiltration, also resulted in reduced infarct size [4]. Alternative approaches, such as FTY720, which anchors cells in lymph nodes, and FK-506 and cyclosporine A, which inactivate nuclear factor of activated T-cells (NFAT), have also shown promising results [4]. Therefore, it seems highly likely that there is an effect of targeting T-cells after an ischemic insult.

T-cell-specific therapies should be administered at the right time according to the temporal infiltration profile and, as suggested by others, targeted against specific T-cell subsets. Jin *et al*. [53] found that both T-helper type 1 (Th1) cells expressing pro-inflammatory cytokines (for example, IL-12 and IFN-γ) and Th2 cells releasing anti-inflammatory cytokines (for example, IL-4 and IL-13) are present in the infarcted brain. In addition, Th2-related cytokines have shown neuroprotective effects and furthermore counterbalance the deleterious Th1 response [6].

Ochs *et al*. [54] examined Th17 cells and regulatory T-cells (Tregs), and concluded that they are pro- and anti-inflammatory, and thereby promote and inhibit tissue damage, respectively. Several studies have shown that Tregs reduce infarct size post-ischemia [2,6,53-55]. Therefore, a more thorough characterization of the temporal profile of various T-cell subsets is definitely needed.

It is concluded by Jin *et al*. [53] that CD8^+^ cytotoxic T-cells contribute significantly to the pathogenesis of ischemia/reperfusion injury. Liesz *et al*. [43] have shown that perforin produced by the cytotoxic T-cells contributes to the ischemic brain damage and Amor *et al*. [16] demonstrated harmful effects of these cells, given that they have cytotoxic abilities *per se*.

It has been found that depletion of γδ^+^-T-cells reduces brain injury post-ischemia and reperfusion, and that γδ^+^-T-cells are a major source of IL-17 [14]. Lakhan *et al*. [2] suggested that targeting these cells might be beneficial following stroke. On the other hand, a study by Kleinschnitz *et al*. [18] concluded that γδ^+^-T-cells play a minor role in infarct development. Thus, the effects of this cell type on infarct development, including a temporal infiltration profile, should be characterized more thoroughly.

Natural killer (NK) T-cells have been found to only have a minor impact on the development of the infarction [18]. However, they have been shown to contribute to a general systemic immunosuppression following stroke by a decline in their activity in the liver [18].

Thus, the general picture of T-cells and their role in stroke is far from clear. However, it seems that the pro-inflammatory Th1 and Th17 cells increase tissue damage, whereas Tregs reduce tissue damage. Therefore, treatment regimens that change the ratios of T-cells in favor of Tregs have the potential to improve the outcome in this patient group. However, as can be seen in Figure 3, T-cell influx is highly time-dependent, suggesting that immunotherapy needs to be initiated at the right time.

### Clinical perspectives

There are few clinical studies regarding inflammatory responses centrally within the brain post-stroke [46,56,57]. Rosell *et al*. [46] examined neutrophilic infiltration in brain sections isolated from stroke patients within 6 hours post-stroke. Immunostaining revealed a strong MMP-9-positive neutrophilic infiltration surrounding brain microvessels, associated with severe basal lamina type IV collagen degradation and blood extravasation. This indicates that neutrophils infiltrate the infarcted brain tissue early post-stroke and are associated with destruction of the blood–brain barrier. Pappata *et al*. [56] used the tracer [C^11^]PK11195 to monitor microglial activity 2 and 24 months following stroke, and found significantly increased binding around the infarcted tissue. In addition, T-cell infiltration as well as dendritic cells has been shown in immunostained brain sections post-stroke [57]. These findings coincide with the studies included in this review, where early neutrophilic infiltration is followed by a later and more sustained infiltration with macrophages/microglia and T-cells.

Regarding peripheral inflammatory responses post-stroke, several studies have found immunosuppressive effects. Sarchielli *et al*. [58] have shown several alterations in the levels of various inflammatory mediators (for example, IL-16, IL-18 and ICAM-1) in stroke patients compared to controls. Furthermore, Li *et al*. [59] and Klehmet *et al*. [50] found a post-stroke reduction in pro-inflammatory cytokine levels as well as CD4^+^ and CD8^+^ T-cells in serum. In general, the data suggest that an overall post-stroke immunosuppression exists in the periphery. Whether this is the result of reduced cell proliferation or increased migration into lymph nodes and tissues, including the brain, as suggested by Yilmaz *et al*. [57], remains to be investigated in detail.

### Limitations of this review

This study is based on data from both rats and mice. Immunological differences as well as similarities between these animals have been shown [60,61]. Examination of the literature reveals immunological differences between specific rat [62-65] as well as mouse strains [66,67]. This issue could have an impact on the analysis and should be taken into account when considering the results of this review. More investigations on human brain tissue, such as the study by Rosell *et al*. [46], are certainly needed, but possess various practical limitations. Nevertheless, the study by Mestas *et al.*[68] found the immunological structure to be quite similar in mice and humans.

## Abbreviations

ANOVA: Analysis of variance; BDNF: Brain-derived neurotrophic factor; FK-506: Tacrolimus; ICAM-1: Intercellular adhesion molecule 1; IFN: Interferon; IL: Interleukin; MCAO: Middle cerebral artery occlusion; MMP: Matrix metalloproteinase; NFAT: Nuclear factor of activated T-cells; NK: Natural killer; pMCAO: Permanent middle cerebral artery occlusion; ROS: Reactive oxygen species; siRNA: Small interfering RNA; Th: T-helper; tMCAO: Transient middle cerebral artery occlusion; tPA: Tissue plasminogen activator; Tregs: Regulatory T-cells; VCAM-1: Vascular cell adhesion molecule 1; WHO: World Health Organization.

## Competing interests

The authors declare that they have no conflicts of interests.

## Authors’ contributions

NVG carried out the literature search and chose the papers included in this review, based on our defined criteria. NVG furthermore drafted the manuscript and carried out the statistical analysis in collaboration with UK. FFJ originally defined the need and purpose of this review and has contributed with fruitful discussions and continuous counseling. UK carried out the statistical analysis and the discussion part in relation to this. HHA supervised the project and has furthermore revised the manuscript. All authors have read and approved the final manuscript.
